# Emergence of terbinafine-resistant *Trichophyton indotineae* in Ontario, Canada, 2014–2023

**DOI:** 10.1128/jcm.01535-24

**Published:** 2024-11-25

**Authors:** Lisa R. McTaggart, Kirby Cronin, Sonja Ruscica, Samir N. Patel, Julianne V. Kus

**Affiliations:** 1 Public Health Ontario 153300 , Toronto, Ontario, Canada; 2 Department of Laboratory Medicine and Pathobiology, University of Toronto 233837 , Toronto, Ontario, Canada; University of Calgary, Calgary, Canada

**Keywords:** *Trichophyton indotineae*, dermatophytosis, terbinafine resistance, antifungal susceptibility, genomic epidemiology, incidence

## Abstract

*Trichophyton indotineae* is an emerging fungal pathogen capable of causing extensive recalcitrant dermatophytosis exacerbated by frequently retained terbinafine resistance. Cases of dermatophytosis due to *T. indotineae* have increased dramatically in India in recent years. Other countries have reported imported infection cases, causing public health concerns over the potential global spread of this drug-resistant pathogen. We describe *T. indotineae* from 47 clinical cases in Ontario, Canada, from 2014 to 2023, based on 50 isolates received by the provincial public health laboratory. Although detected in Ontario as early as 2014, cases increased sharply in 2022–2023. The majority of cases were found in 20 to 64-year-olds (83.0%). Cases from 2022 to 2023 were noted in only 8 of 34 public health units, clustering in large urban centers. Based on genomic epidemiology, Ontario isolates were genetically similar to isolates from other countries. Neither temporal nor geographic clustering based on country or public health unit was observed, suggesting Ontario cases represented recent introductions from endemic regions, although limited local transmission cannot be ruled out. Genome diversity (4.4 × 10^−6^) was extremely low, consistent with the hypothesized recent emergence and clonal population structure of *T. indotineae*. Most (71.4%) cases were terbinafine-resistant, with isolates exhibiting either the L393F/L393S (*n* = 3) or F397L (*n* = 27) squalene epoxidase substitution. Decreased susceptibility to itraconazole or voriconazole was found among 23.7% of isolates and was frequently associated with *CYP51B* gene duplication. Terbinafine-resistant *T. indotineae* infections are escalating in Ontario, meriting enhanced laboratory detection methods and public health efforts to increase the awareness of recalcitrant dermatophytoses in order to promptly initiate appropriate treatment and control transmission.

**IMPORTANCE:**

Cases of dermatophytosis caused by emerging pathogen *Trichophyton indotineae* are increasing worldwide. Many are resistant to first-line treatment option terbinafine, resulting in difficult-to-treat cases. We describe the emergence of cases of *T. indotineae* infections in Ontario, Canada. The incidence in primarily urban centers increased dramatically in 2022–2023, with a large percentage of isolates resistant to terbinafine. Decreased susceptibility to azoles was also observed for some isolates, raising concern over the potential evolution of multi-drug resistance. Ontario *T. indotineae* isolates were genetically similar to those from disparate locales worldwide, signifying the global nature of this public health concern. Together with other reports, this study serves to raise public health awareness to promote better laboratory detection procedures, prompt appropriate treatment of recalcitrant dermatophytoses, and inform infection prevention and control measures.

## INTRODUCTION

Dermatophytoses are superficial mycoses of the skin, hair, and nails caused by several species of keratinophilic fungi including *Trichophyton rubrum*, *Trichophyton interdigitale,* and other *Trichophyton* spp., as well as *Microsporum* spp. and *Epidermophyton* spp. (1). While not fatal, these infections are highly prevalent, affecting 20%–25% of the global population, causing severe morbidity due to uncomfortable inflammation, itchy rashes and scaling at superficial body sites, and superinfections (1, 2). Nevertheless, dermatophytoses have historically been regarded as a relatively low-priority clinical and public health problem, in part because effective treatment with a variety of antifungals is available without prescription in many countries (3).

Recently, a newly described species *Trichophyton indotineae* (previously *Trichophyton mentagrophytes* VIII) has been reported to cause cases of extensive, recalcitrant tinea cruris and tinea corporis (4–6). Over the past decade, an alarming increase in the incidence of *T. indotineae* infections has been reported in India such that in a recent survey, the vast majority (78%) of dermatophyte infections in this country were found to be caused by *T. indotineae* (7). Now considered endemic to India and Iran (5, 8), many other countries are reporting imported cases of *T. indotineae* with possible rare cases of local transmission (2, 5, 6, 9–13), signaling the potential for this epidemic to spread to additional countries in the near future. Many cases of *T. indotineae* dermatophytosis are recalcitrant to both topical and oral preparations of first-line treatment option terbinafine because a large number of isolates demonstrate *in vitro* elevated MICs to terbinafine (MIC ≥0.2 µg/mL) due to point mutations causing amino acid substitutions in squalene epoxidase (SQLE) (5, 7, 10, 14, 15). While there are currently no clinical breakpoints, MICs ≥ 0.2 µg/mL are thought to reflect resistance (16). In fact, it is postulated that *T. indotineae* emerged as an anthropophilic, clonal offshoot from zoophilic strains of *T. mentagrophytes* due to the overuse of over-the-counter topical corticosteroid–antifungal combinations, allowing for the undeterred spread of resistant strains (3). While closely related to other members of the *T. mentagrophytes* complex, *T. indotineae* can be screened for by negative urease and hair perforation tests; however, currently, species-level identification requires internal transcribed spacer (ITS) and translation elongation factor (TEF) sequence analysis (4). Matrix-assisted laser desorption ionization time-of-flight mass spectrometry (MALDI-ToF MS) may also have the potential to identify this organism to species level (17, 18).

Here, we describe *T. indotineae* from cases in Ontario, Canada, from 2014 to 2023 including infection trends, geographic incidence, genomic epidemiology, and antifungal susceptibility. Ontario is Canada’s most populous province, comprising ~40% of the country’s total population (19). It is also home to many immigrant communities of diverse nationalities with frequent travel to their respective countries of origin, including countries endemic to *T. indotineae*. Therefore, we provide a timely update to document the presence of *T. indotineae* in a region of North America with strong international ties in order to raise public health and laboratory awareness. We hope to prompt more effective interventions, including treatment options, and limit the spread of this emerging infectious disease.

## MATERIALS AND METHODS

A total of 47 cases represented by 50 T. *indotineae* isolates were included in the study. Three cases were represented by duplicate samples from the same patient. Study samples included 41 fungal culture isolates identified as *Trichophyton indotineae* derived from either the primary specimen or referred cultures from 38 individual patients submitted to Public Health Ontario (PHO) Laboratory, the reference microbiology laboratory for Ontario, from 01 January 2022 to 31 December 2023. Isolates were provisionally identified as *T. indotineae* by negative urease test and confirmed by internal transcribed spacer 2 (ITS2) and TEF sequence analysis (3, 4). Additionally, we included nine cases from 2014 to 2021 retrospectively identified as *T. indotineae* by re-analysis of historical *Trichophyton* ITS2 sequences. Of the 50 T. *indotineae* isolates, eight were not archived and therefore unavailable for subsequent experimentation (one isolate each from 2014, 2016, and 2017 and five isolates from 2023).

ITS2 and TEF PCR and sequencing were carried out using HotStar Taq polymerase master mix (Qiagen) and primers ITS3 (5′-GCATCGATGAAGAACGCAGC-3′), ITS4 (5′-TCCTCCGCTTATTGATATGC-3′) (20), and EF1α-F (5′-CACATTAACTTGGTCGTTATCG-3′) EF1α-R (5′-CATCCTTGGAGATACCAGC-3′) (3). PCR reactions were cycled at 95°C for 15 minutes, 35 cycles of 94°C for 30 seconds, 52°C for 30 seconds, 72°C for 1 minute, followed by a final extension of 72°C for 10 minutes. Sanger sequencing was performed using BigDye v3.1 on an Applied Biosystems 3500 genetic analyzer as per the manufacturer’s instructions (Thermo Fisher Scientific). For identification purposes, TEF and ITS2 sequences were compared to those of *T. indotineae* DSM 107596 (GenBank accession MH802490) (10) and CBS 146623 (GenBank accession NR_173767) (4) respectively.

The incidence of *T. indotineae* cases per 100,000 population per year (2022–2023) was calculated using annual population denominators obtained from IntelliHealth Ontario (21). For geographic analysis and mapping, *T. indotineae* cases were assigned to a Public Health Unit (PHU) based on the patient’s residential postal code, if known, or alternatively based on the submitter’s postal code. Maps were generated using the custom Public Health Ontario developed mapping program Easy Maps v2.0.

Next-generation sequencing libraries were prepared for 42 isolates from DNA extracted with the Qiagen PowerSoil Pro kit (Hilden, Germany) using the Nextera XT library preparation kit (Illumina, San Diego, CA, USA) and sequenced on Illumina MiSeq v2 or NextSeq 550 mid-output, 300-cycle cartridges as per the manufacturer’s protocol (Illumina).

Single-nucleotide variant (SNV) analysis was conducted using the MycoSNP-nf analysis pipeline (22) using *T. indotineae* TIMM20114 as the reference genome (GCA023065905.1) with a sample ploidy of 1 (23). In addition to the 42 Ontario isolates, we included raw fastqs downloaded from NCBI SRA for six isolates from India (SRR16944738, SRR16944739, SRR16944740, SRR17381637, SRR17381638, and SRR17381639) (PacBio long-read data) (23), seven isolates from two patients in Singapore (SRR26405630, SRR26405631, SRR26405632, SRR26858049, SRR26858050, SRR26858051, and SRR26858052) (Illumina short-read data) (24), and 11 isolates from New York, USA (SRR27198731 to SRR27198741) (Illumina short-read data) (25). Prior to MycoSNP processing, minimap2 was used to align the PacBio reads to the reference genome, which had previously been processed by the MycoSNP pipeline to mask repetitive regions. Aligned BAM files were processed with HaplotypeCaller from GATK v 4.5.0.0 (26) to generate gVCF files for input into the MycoSNP pipeline using the –add_vcf_files option. Phylogenomic analysis of whole-genome SNVs (single-nucleotide variants) was conducted in MEGA11 (27) using the neighbor-joining method with the pairwise deletion model and 500 bootstrap replications. DNA sequences for regions for SQLE (JAJVHL010000003.1: 941062–942593) and sterol 14-α demethylase genes *CYP51A* (JAJVHL010000002.1:3271311–3272943) and *CYP51B* (JAJVHL010000003.1:2121925–2123712) were generated using the GATK FastaAlternateReferenceMaker tool and interrogated for amino acid substitutions compared to wild-type sequences (5, 16, 28). The average nucleotide diversity (π) for 59 isolates representing unique cases was calculated using the PopGenome package (29) in R v4.3.3 and normalized for the reference assembly size. Additionally, isolate DNA was tested for *CYP51B* gene duplication by PCR assays targeting the region between tandem duplicates, as previously described (30).

Antifungal susceptibility testing (AFST) for 38 isolates was performed by broth microdilution according to the Clinical Laboratory Standards Institute (CLSI) guidelines (31, 32) using custom panels (ThermoFisher, Waltham MA, USA). Isolates (*n* = 2) representing patient duplicates were not tested. When cultured on potato dextrose agar, two isolates failed to sporulate; therefore, AFST panels were not able to be set up. Because the broth microdilution panels did not contain terbinafine, isolates (*n* = 42) were screened for their ability to grow on sabouraud dextrose agar (SDA) containing 0.2 µg/mL of terbinafine compared to a drug-free control SDA plate (16).

This investigation was conducted as part of the PHO’s legislated mandate to provide scientific and technical advice as well as operational support in emergency or outbreak situations (33), c 10) (33). As this work is considered public health practice and not research, research ethics approval was not required.

Specimens and associated data were anonymized prior to use by a PHO data custodian, and accordingly, individual consent was not required for the secondary use of nonidentifiable specimens and associated information.

## RESULTS

**Fig 1:**
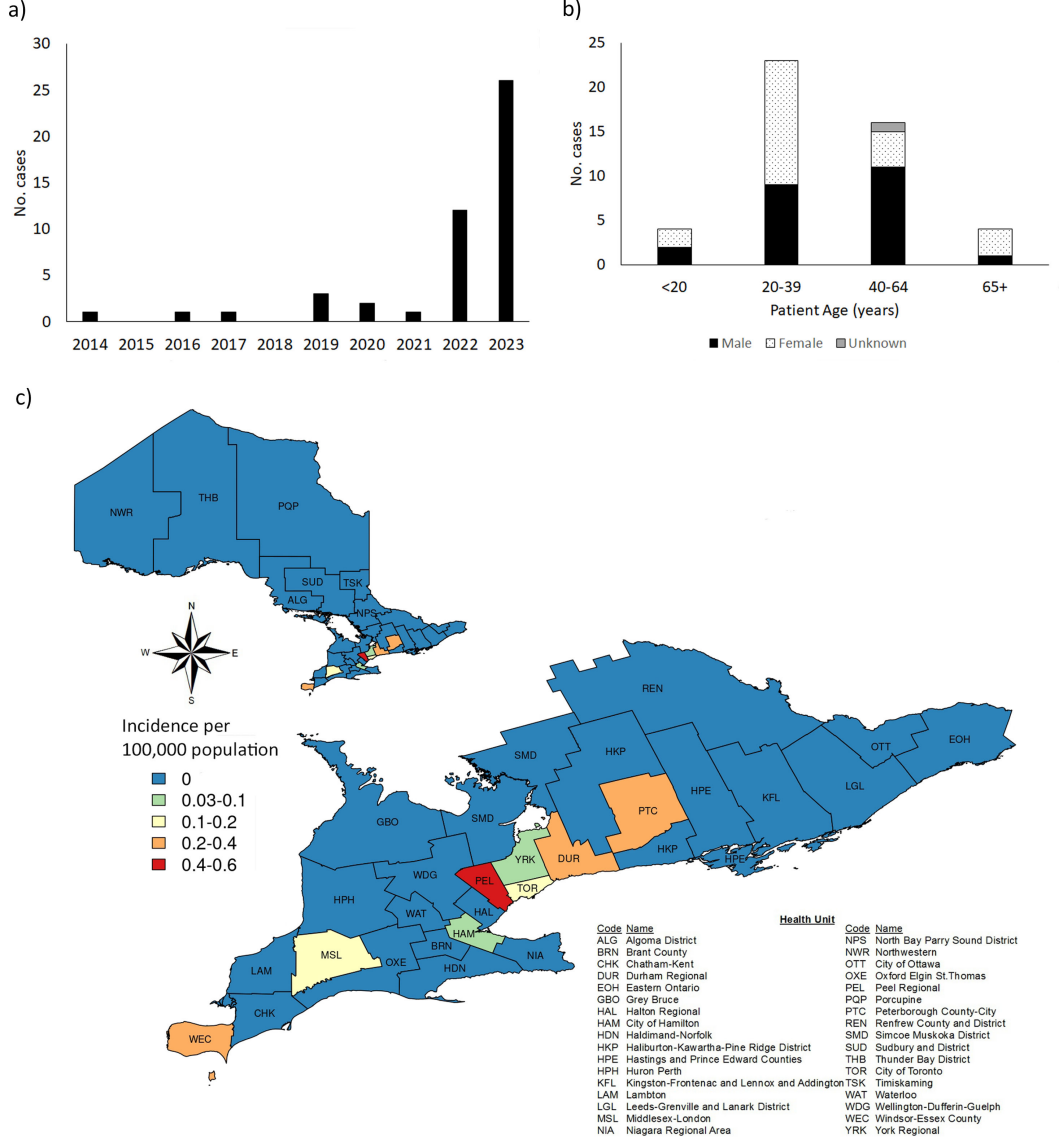
(a) Number of laboratory-confirmed cases of *T. indotineae* in Ontario per year from 2014 to 2023. (b) Patient age and sex of laboratory-confirmed cases of *T. indotineae,* and (c) incidence of laboratory-confirmed cases of *T. indotineae* per 100,000 population per year for 2022–2023 (*n* = 38 cases) in Ontario Public Health Units.

A total of 47 cases of dermatophytosis due to *T. indotineae* were identified by PHO Laboratory in Ontario from 2014 to 2023 from 50 individual specimens/isolates received in our laboratory. Of the 47 cases, 38 were identified based on 100% similarity to *T. indotineae* ITS2/TEF sequences, and negative urease tests were from 2022 to 2023 compared to nine cases prior to 2022, which were identified retrospectively by ITS2 sequence analysis alone (Fig. 1a). All isolates (*n* = 50) were recovered from skin with the body site specified for 33 isolates, including the torso (abdomen, back, breast, buttock, and groin, *n* = 15), arm/hand (*n* = 3), leg/foot (*n* = 13), or head/neck (*n* = 2); skin: site not specified (*n* = 17). Cases were derived from 48.9% male patients (23/74) encompassing a wide patient age range (13–80); however, the majority of cases (83.0%) were recovered from adults aged 20–64 years old (Fig. 1b). Of 34 local Public Health Units across the province, cases of *T. indotineae* were identified in only eight (Fig. 1c) and were primarily clustered in large urban centers of the province.

**Fig 2:**
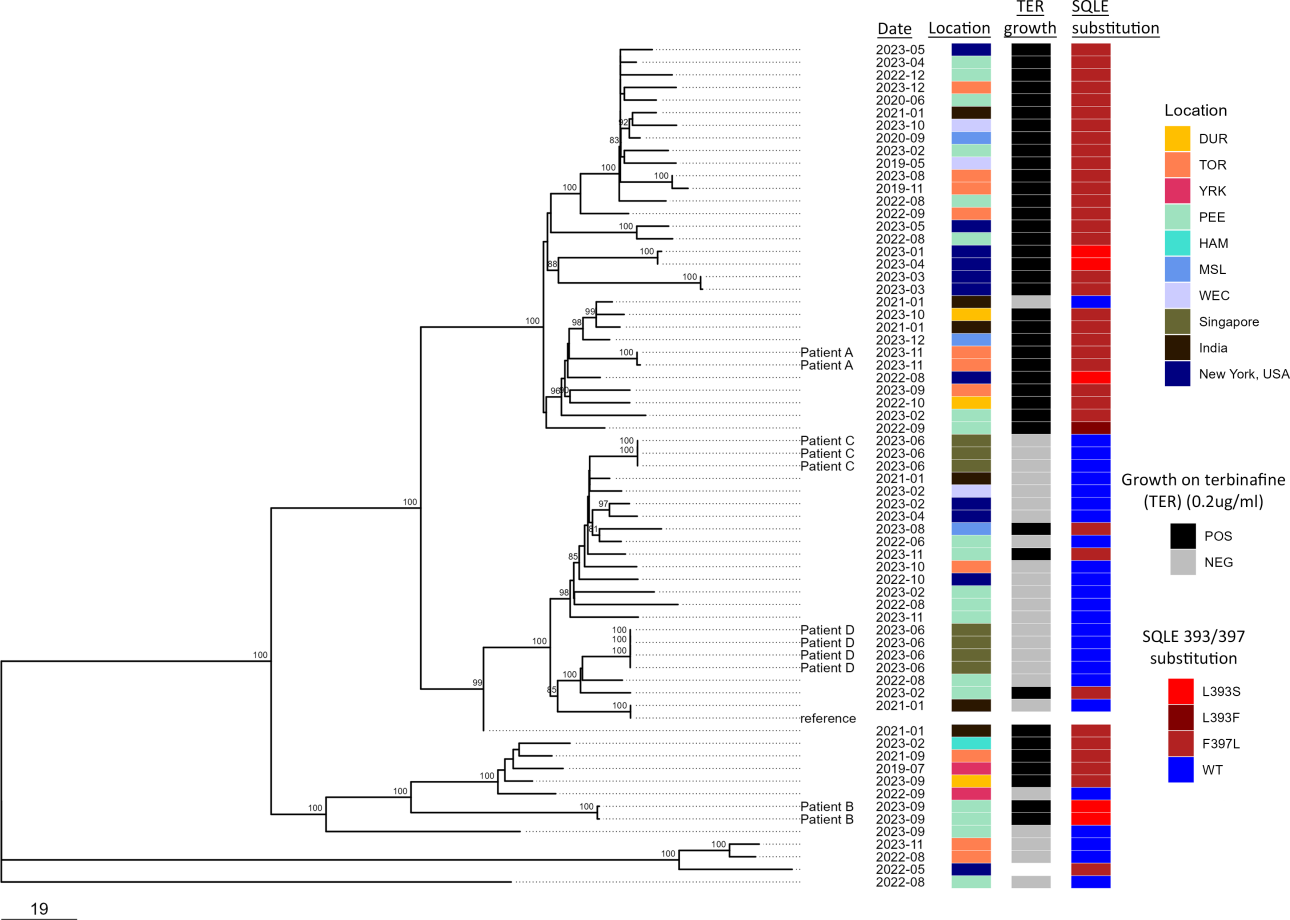
Phylogenomic analysis of whole-genome SNVs of *T. indotineae* isolates by the neighbor-joining method. The percentage of trees of 500 bootstrap replications in which the associated taxa clustered together is shown next to the nodes. The tree is drawn to scale, with branch lengths measured in the number of substitutions per site. There were a total of 1,341 positions in the final data set of 67 sequences. The isolates are labeled by the date the specimen was received at PHOL or date collected as indicated on the SRA record for isolates from India (23), Singapore (24), or New York, USA (25). Duplicate isolates from the same patient were available for two Ontario patients, Patient A (PHO44539 and PHO44795) and Patient B (PHO44592 and PHO44593), as well as two patients from Singapore, Patient C (Ti725_1, Ti725_2, and Ti725_3) and Patient D (TID1a_1, TID1_1, TID2_1, and TID2a_1) (24). For Ontario isolates (*n* = 42), the Public Health Unit (as defined in Fig. 1c) in which the patient or alternatively submitter was located is shown. For non-Ontario isolates (*n* = 24), the country of origin is indicated. The presence of growth on 0.2 µg/mL terbinafine (TER) and SQLE substitutions is indicated.

Phylogenomic analysis of whole-genome SNVs demonstrated that Ontario isolate sequences (*n* = 42) were interspersed among isolate sequences from India (*n* = 6) (23), seven isolate sequences from two patients representing cases imported from India to Singapore (24), and 11 isolate sequences from patients in New York, USA (25) (Fig. 2). Neither geographic nor temporal clustering based on the country or Ontario Public Health Unit was observed. As expected, isolate sequences from the same patient (Patients A–D) were nearly identical with 0–1 SNVs between sequences (Fig. 2). SNV distances between isolate sequences from unique cases ranged from 0 to 375 SNVs with an average of 121. The average nucleotide diversity normalized for reference genome size was 4.4 × 10^−6^.

Since terbinafine is the first-line treatment option for dermatophyte infections (2), we screened *T. indotineae* isolates (*n* = 42) for their ability to grow on SDA containing 0.2 µg/mL terbinafine. This media was previously used to successfully screen for resistant *T. indotineae* (16) and aligns with the EUCAST terbinafine tentative epidemiological cutoff value (ECV) of 0.125 mg/L for *T. indotineae* (34). Growth on terbinafine-containing media was observed for 71.4% (30/42) of Ontario isolates, which correlated with either the L393F or L393S substitution (*n* = 3) or the F397L substitution (*n* = 27) in squalene epoxidase (SQLE) known to mediate high MICs to terbinafine associated with treatment failure (5) (Fig. 2; Table S1). The 28.6% (12/42) of isolates that failed to grow on terbinafine possessed the wild-type SQLE (Fig. 2; Table S1). Based on the phylogenomic analysis, some clades possessed disproportionately more isolates capable of growth on terbinafine-containing media than others (Fig. 2).

**Fig 3:**
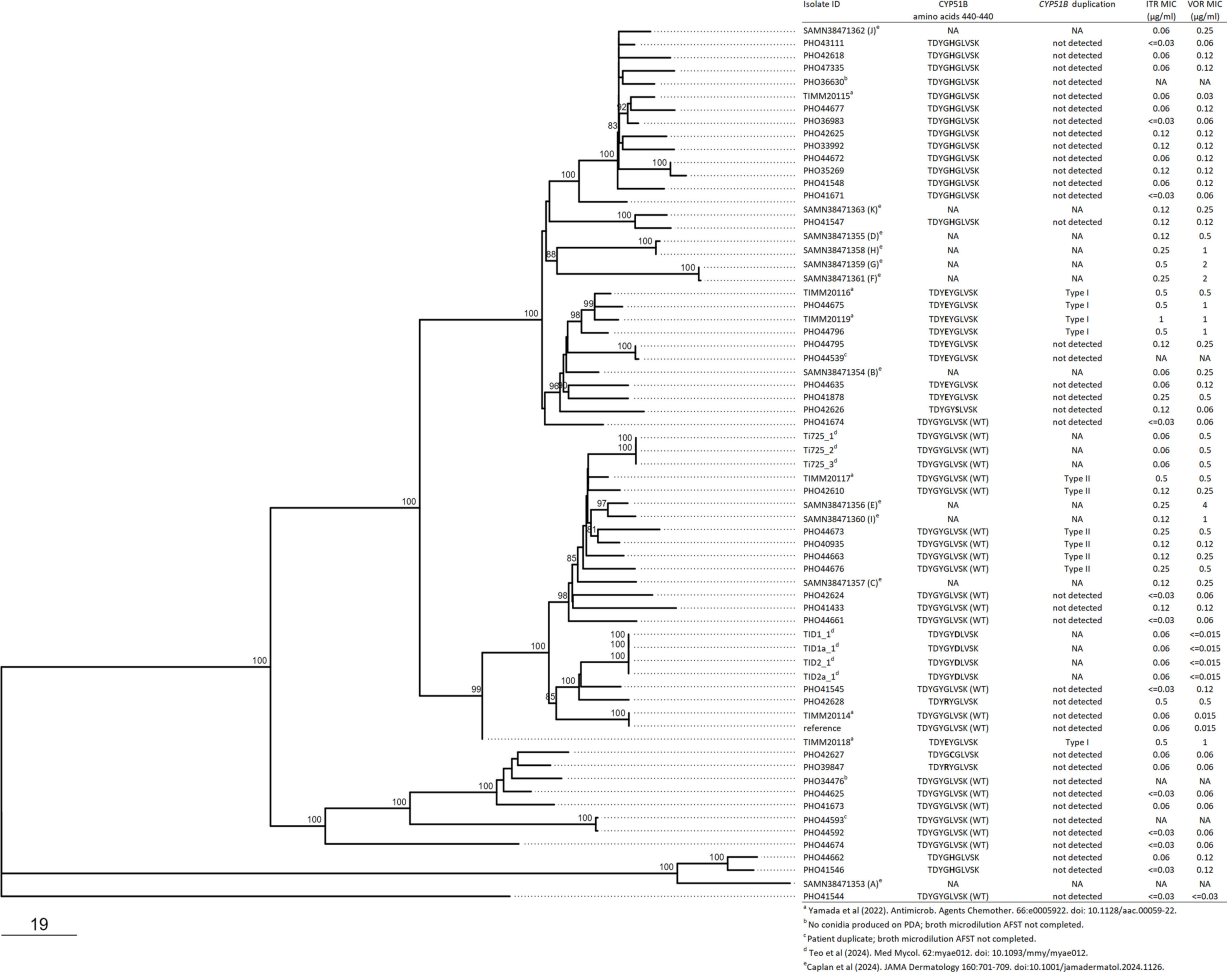
SNV phylogenomic tree from Fig. 2 together with antifungal susceptibility data including CYP51B 440–449 amino acid sequence, presence of *CYP51B* gene duplication, and MICs to itraconazole (ITR) and voriconazole (VOR) tested by the broth microdilution method.

Broth microdilution antifungal susceptibility profiles of all tested (*n* = 38) Ontario *T. indotineae* isolates demonstrated low MECs (minimum effective concentration) to the anidulafungin (MEC_90_ ≤0.015), caspofungin (MEC_90_ ≤0.03), and micafungin (MEC_90_ ≤0.015) (Table S1). Based on ECVs of MIC ≥0.25 µg/mL to voriconazole and/or MIC ≥0.5 µg/mL to itraconazole (7, 10, 35), 9 of 38 (23.7%) isolates exhibited decreased (non-wild-type) susceptibility to itraconazole and/or voriconazole (Fig. 3; Table S1). Since decreased susceptibility to azoles has been attributed to amino acid substitutions in sterol 14-α demethylase encoded by *CYP51A* and *CYP51B* in filamentous fungi such as *A. fumigatus* (36) and *CYP51B* gene duplication in *T. indotineae* (23, 30), we screened Ontario isolates for these elements (Fig. 3; Table S1). No amino acid substitutions were detected in CYP51A sequences; however, several different amino acid substitutions were detected in CYP51B but with no apparent correlation with azole MIC (Fig. 3; Table S1). Among 38 Ontario isolates, both Type I (*n* = 2) and Type II (*n* = 5) *CYP51B* gene duplication events were detected by PCR assay (30), and these isolates clustered together phylogenomically with isolates from India exhibiting Type I and Type II *CYP51B* gene duplications, respectively (Fig. 3; Table S1). The majority (six of seven) of *T. indotineae* isolates with Type I or Type II *CYP51B* gene duplications displayed decreased (non-wild-type) susceptibility to itraconazole (MIC ≥0.5 µg/mL) or voriconazole (MIC ≥0.25 µg/mL) with MICs for isolates with Type I *CYP51B* gene duplication higher than those with Type II duplication events (Fig. 3; Table S1). Also, a single isolate (40935) had a Type II *CYP51B* gene duplication but with MICs to itraconazole and voriconazole below the respective ECVs (Fig. 3; Table S1). Conversely, three isolates (41878, 44795, and 42628) had MICs greater than or equal to the ECVs for itraconazole or voriconazole, but neither Type I nor Type II *CYP51B* gene duplication events were detected (Fig. 3; Table S1). We also noted a concomitant increase in MICs for posaconazole and isavuconazole in isolates with MICs greater than or equal to the ECVs for itraconazole and/or voriconazole (Table S1). Interestingly, the MIC_50_ and MIC_90_ values for isavuconazole were 2 or 3 twofold dilutions higher than those for vorizonazole, itraconazole, and posaconazole (Table S1), which has been observed by others (S. Chaturvedi, unpublished data).

## DISCUSSION

Here, we describe cases of dermatophytosis due to *T. indotineae* in Ontario, Canada, through to 2023. *T. indotineae* is easily transmitted person-to-person through close contact and via fomites (37); sexual transmission has been reported as well (38). While a few probable cases were noted in the province as early as 2014, the vast majority were identified in 2022 and 2023, coinciding with both the rapid global spread of *T. indotineae* (5, 6, 8) and the lifting of COVID-19 travel restrictions in Canada. Cases of dermatophytosis due to *T. indotineae* have also been identified among isolates from 11 US states and two Canadian provinces submitted to a large reference testing laboratory (2021–2022) (12), in New York (2021–2023) (25, 39) and in other laboratories in Ontario (2021) (13). There was a notable absence of nail specimens typical of other dermatophytoses and a preponderance of skin specimens from torso samples, typical of *T. indotineae* infections (tinea corporis and tinea cruris) (3, 13, 39). Cases primarily occurred in adults of both sexes (8).

Genomic epidemiological analysis demonstrated that *T. indotineae* isolates in Ontario clustered genetically with isolates from other countries, including India (23), Singapore (with suggested importation from India) (24), and New York, USA (with likely importation from Bangladesh) (25). The analysis precludes the presence of an Ontario-specific strain, suggesting that as of 2023, Ontario cases were largely imported, although limited local transmission cannot be ruled out. Individual case travel histories were not available to us, so potential local transmission cannot be assessed as was postulated to have occurred with cases in New York City (25, 39). *T. indotineae* is endemic to India (7) and Iran (5), with many other countries reporting imported cases rather than local transmission (5, 6, 9). Ontario is home to a large multinational population with frequent travel to respective countries of origin (40). Concordantly, cases of *T. indotineae* in Ontario predominated in large urban centers, such as the Greater Toronto Area, where many immigrants reside (40).

*T. indotineae* is postulated to have emerged as a recent anthropophilic clonal offshoot of *T. mentagrophytes* (3). In previous studies, high mobility group (HMG) domain transcription factor gene corresponding to mating type (+) was detected among all *T. indotineae* strains examined with a few isolates displaying both HMG and α-box genes, suggesting a clonal outbreak population structure and perhaps a loss of sexuality in this species (3, 14). Consistent with this hypothesis, we observe a low level of divergence among all isolates in our study. SNVs were detected at only 1,341 positions along the reference genome; 99.99% of positions were identical. On average, isolates differed by only 121 SNVs, similar to the low level of diversity reported elsewhere (14). The genome diversity of our *T. indotineae* data set was 4.4 × 10^−6^, which is 2 and 3 orders of magnitude lower than another largely clonal dermatophyte *T. rubrum* (5.4 × 10^−4^) (41) or the actively recombining fungal pathogen *Cryptococcus neoformans* var. *grubii* (7.4 × 10^−3^) (42), respectively. The low level of genetic diversity supports the hypothesis that *T. indotineae* possesses a clonal population structure.

Terbinafine is a first-line treatment option for dermatophytosis. Similar to several other reports from India (7, 14, 25), a large percentage (71.4%) of *T. indotineae* isolates in this study exhibited decreased *in vitro* susceptibility to terbinafine, which has also been associated with treatment failure (5, 25). This is in line with a recent report from a large North America reference laboratory where 18 of 21 (85.7%) *T*. *indotineae* had elevated MICs to terbinafine ≥0.2 µg/mL (12). While this is a cause for concern, it is expected that a greater number of specimens from recalcitrant cases of tinea corporis and tinea cruris were submitted to PHO Laboratory for additional testing than from treatable cases. Thus, the percentage of isolates (71.4%) reported here exhibiting decreased susceptibility to terbinafine may overestimate the level of terbinafine resistance among *T. indotineae* in Ontario. All isolates demonstrating growth on 0.2 µg/mL terbinafine also possessed amino acid substitutions at position 393 or 397 of SQLE of the ergosterol synthesis pathway, which is strongly associated with terbinafine resistance among isolates in other studies (5, 7, 10, 14, 15, 25). Phylogenomic clustering of isolates with decreased susceptibility to terbinafine suggests that human-to-human transmission of resistant isolates is likely contributing to the increase in recalcitrant cases of dermatophytosis, in addition to *de novo* mutations acquired due to selective pressure as a result of overuse of topical corticosteroid–antifungal treatments (3).

While AFST MECs toward the echinocandins were low, suggesting that *T. indotineae* are susceptible to micafungin, caspofungin, and anidulafungin *in vitro*, the clinical efficacy and practicality of treatment with these intravenously delivered drugs are uncertain. Itraconazole is reported as an effective treatment option for terbinafine-resistant cases (8, 25, 39); however, non-wild-type MICs to voriconazole and itraconazole elevated above ECVs of ≥0.25 µg/mL and ≥0.5 µg/mL, respectively, were detected among some isolates in this study and elsewhere (23, 30). Substitutions in the hotspot regions of CYP51, a component of the ergosterol synthesis pathway, have been implicated in azole resistance in *Aspergillus fumigatus* (36). However, as reported here and in other studies, there appears to be no correlation between CYP51B substitutions in positions 441–444, corresponding to the hot spot region, and elevated azole MICs in *T. indotineae* (23, 28). Additionally, another study (23) demonstrated that although a G443E substitution was observed in CYP51B of some *T. indotineae* isolates, replacement of *CYP51B* with an allele encoding a G443E substitution in *T. mentagrophytes* did not alter the itraconazole or voriconazole MICs, suggesting that such CYP51B amino acid substitutions do not confer a selection advantage to *Trichophyton* spp. in the presence of azoles. Conversely, elevated MICs to itraconazole and voriconazole have been attributed to two different types of *CYP51B* tandem gene duplication, resulting in overexpression of *CYP51B* in *T. indotineae* (23, 30). Both Type I and Type II *CYP51B* gene duplications were detected among Ontario isolates and were correlated with decreased susceptibility to azoles. Phylogenomic clustering of isolates with Type I and Type II *CYP51B* gene duplication, respectively, suggests relatedness by evolutionary descent facilitated by human-to-human transmission. There were three isolates with elevated MICs to itraconazole and voriconazole, where neither Type I nor Type II *CYP51B* gene duplications were detected. It is possible that other mechanisms including other *CYP51B* gene duplication configurations, in addition to Type I and Type II, exist in these isolates. As well, a single isolate had a Type II *CYP51B* gene duplication but maintained low MICs to itraconazole and voriconazole. This phenomenon was observed previously (30), and it is possible that *CYP51B* expression, not tested in this study, was not elevated in this isolate, despite the *CYP51B* gene duplication.

This study is subject to limitations. Case ascertainment underestimates the true burden of infection, especially prior to 2022, due to challenges with species identification owing to the fact that this is a newly described species and an emerging public health concern. Specifically, there were likely *T. indotineae* infections successfully treated with over-the-counter medications that were not included in this study because they were not forwarded to PHO for investigation. Since no CLSI breakpoints exist for antifungals toward *T. indotineae*, it is unclear whether the MICs determined in this study are likely to result in treatment failure. Individual case travel data and contact tracing were not available for this study, so the possibility of local transmission cannot be determined. Despite the many reports citing *T. indotineae* infections as an emerging global health concern, there are few publically available WGS data sets to aid in contextualizing the diversity and population structure observed among Ontario isolates.

In conclusion, recent cases of drug-resistant *T. indotineae* dermatophytosis have been identified in Ontario, Canada; however, phylogenomic analysis suggests that these cases are genetically similar to cases from India and, at this time, likely represent imported cases. Although breakpoints for antifungal drugs do not exist, elevated MICs among some isolates to terbinafine, itraconazole, and voriconazole allude to possible ineffective treatment with these antifungals, and anecdotally several of these isolates were specifically sent to our lab for testing because patients were failing to respond to therapy. As with *T. rubrum* (43), low MECs suggest excellent *in vitro* activity of the echinocandins against *T. indotineae* isolates; however, additional investigation is needed to determine the clinical efficacy. This study serves to heighten the awareness of this emerging global public health concern in a North American jurisdiction experiencing significant immigration and global travel. We call for increased vigilance among physicians to refer cases of recalcitrant dermatophytosis for enhanced investigation and analysis of appropriate treatment options.

## Data Availability

Raw fastq data are available in BioProject PRJNA1149028, with individual sample Sequence Read Archive numbers listed in Table S1.

## SUPPLEMENTAL MATERIAL

Table S1 jcm.01535-24-s0001.xlsx Characteristics of study isolates.

ASM does not own the copyrights to Supplemental Material that may be linked to, or accessed through, an article. The authors have granted ASM a non-exclusive, world-wide license to publish the Supplemental Material files. Please contact the corresponding author directly for reuse.
